# Uncovering the Dynamic System Driving Older Adults' Vitality: A Causal Loop Diagram Co‐Created With Dutch Older Adults

**DOI:** 10.1111/hex.70344

**Published:** 2025-07-09

**Authors:** Djoeke Besselink, Fons van der Lucht, Lisa Barsties, Martine Jansen‐ van der Vliet, Thomas G. Kuijpers, Lidwien Lemmens, Evelyn J. Finnema, Saskia W. van den Berg

**Affiliations:** ^1^ Regional Public Health Service, GGD Groningen Groningen the Netherlands; ^2^ FAITH research Leeuwarden the Netherlands; ^3^ Research Group Healthy Ageing, Allied Health Care and Nursing Hanze University of Applied Sciences Groningen the Netherlands; ^4^ Health Science‐Nursing Science and Education University Medical Center Groningen, University of Groningen Groningen the Netherlands; ^5^ Centre for Public health, Healthcare and Society National Institute for Public Health and the Environment (RIVM) Bilthoven the Netherlands; ^6^ Centre for Prevention, Lifestyle and Health National Institute for Public Health and the Environment (RIVM) Bilthoven the Netherlands; ^7^ Research Group Living, Wellbeing and Care for older people NHL Stenden University of Applied Sciences Leeuwarden the Netherlands; ^8^ Research Group Nursing Diagnostics Hanze University of Applied Sciences Groningen the Netherlands

**Keywords:** causal loop diagram, co‐creation, group model building, older adults, systems thinking, vitality

## Abstract

**Background:**

Most studies on older adults' vitality focus on linear connections between determinants and outcomes. To design more comprehensive and impactful approaches to support the vitality of older adults, a better understanding of the interplay among elements that shape their vitality is necessary.

**Objective:**

To uncover the underlying dynamic system that drives vitality in older adults, drawing directly from older adults' perspectives.

**Methods:**

During three group model‐building sessions with 10–12 older adults (≥ 55 years old), a causal loop diagram with relevant feedback loops was developed through co‐creation with older adults. The construction and analysis of the causal loop diagram were facilitated using the online modelling tools Vensim and Kumu. The group model‐building sessions were guided by Scriptapedia, an online guide to conducting group model‐building practices.

**Results:**

The final CLD consisted of 15 elements contributing to older adults' vitality, organised into three themes: ‘Psychological and emotional elements’, ‘Social connections and support’ and ‘Lifestyle and habits’. A total of 41 reinforcing feedback loops were identified, with 21 involving all three themes, 15 connecting two themes and 5 within a single theme.

**Conclusions:**

This study displays the complex interplay of elements influencing older adults' vitality, highlighting the critical roles of psychological, social and lifestyle‐related elements. The participatory‐led approach yielded co‐produced insights that inform public health strategies, underscoring the need for comprehensive, multidisciplinary approaches to promote older adults' vitality. Such approaches must offer social opportunities and foster individuals' capacity and motivation to engage in meaningful social relationships.

**Patient or Public Contribution:**

The study was conducted in collaboration with a municipal policymaker and a coordinator of local community centres, who provided input on participant recruitment, materials, data interpretation, ethical considerations and result dissemination. During data collection, twelve older adults participated in three group model‐building sessions, collaboratively developing a causal loop diagram to explore elements of vitality and their interconnections. Ongoing member checking with participants throughout the process ensured the analysis was refined and the researchers' interpretations were validated.

## 1 Introduction

Shifts in public health policies, advanced medical practice and improvements in social and economic conditions have contributed to increased longevity in European countries [1]. In the Netherlands, the number of people aged 65 years and over is projected to reach approximately 4.8 million by 2040, representing a quarter of the Dutch population [2]. This demographic shift poses major public health challenges, including workforce shortages and rising healthcare costs, which threaten the quality and accessibility of care for older adults [3, 4]. To ensure the sustainability of the healthcare system, Dutch public health strategies aim to keep older adults independent for as long as possible through the promotion of their vitality [5].

Vitality is a multifaceted construct that integrates physical, psychological and social dimensions of health and well‐being [6, 7]. At an individual level, vitality can be understood as a state of ‘being able to do what you want to do’, allowing people to pursue their goals and personal desires while effectively managing the challenges that arise with ageing [6, 8]. By enhancing vitality, older adults are better equipped to navigate the complexities of ageing, such as functional decline and health issues, while continuing to engage actively in their communities and personal lives. Therefore, promoting vitality among older adults presents significant opportunities to support and empower them to maintain their independence for as long as possible [6, 8, 9, 10].

However, promoting older adults' vitality is not a straightforward endeavour. Challenges stem from the complex and subjective nature of vitality, leading to considerable variation in how individuals experience and interpret it [7, 9]. While qualitative studies can provide valuable insights into how older adults perceive their vitality, such research remains scarce. Most existing studies rely heavily on linear, quantitative measures, which may overlook the complex, non‐linear relationships among the various elements contributing to vitality [7, 11]. Consequently, strategies to promote older adults' vitality tend to undermine its integrative, dynamic nature [6]. To address these practical challenges, a better understanding of the dynamic interplay between elements contributing to older adults' vitality is needed.

Systems thinking is an approach to understanding complex issues. Rather than viewing factors in isolation, systems thinking examines patterns, feedback loops and relationships within a system to uncover how certain outcomes, such as vitality, are produced and sustained. This perspective is particularly useful when addressing multifaceted constructs because it offers a framework for capturing and understanding the dynamic interplay among the various dimensions of vitality [12, 13, 14]. Group model building (GMB) is a qualitative, participatory method grounded in systems thinking. It fosters dialogue and co‐learning among participants, allowing them to share their perspectives and experiences. Through structured GMB sessions, these diverse insights are combined to co‐create a visual representation of how different factors interact dynamically within the system [15]. This methodology enhances the exploration of older adults' vitality through a comprehensive, real‐world lens, which is crucial for developing effective interventions and policies targeting vitality.

To the best of our knowledge, no prior research has examined the elements contributing to older adults' vitality through a participatory systems approach. Therefore, this study aims to uncover the dynamic system underlying older adults' vitality by drawing directly from their perspectives. We focused on adults aged 55 years and over to capture a broader range of experiences and insights, acknowledging that the transition into later life can shape how people perceive and manage their health and well‐being [16]. The guiding research question was: ‘What elements contribute to the vitality of older adults (aged 55 and over), and how do these elements interrelate?’.

## 2 Methods

### 2.1 Design

Through GMB, older adults (≥ 55 years old) and researchers co‐created a causal loop diagram (CLD) on the elements contributing to older adults' vitality. The research team consisted of experts in (public) health research and GMB, all specialising in the health and care needs of older adults.

A CLD is a visual tool used to map feedback loops and causal relationships within a complex system [17]. Feedback loops represent the cyclical relationships between elements operating in a system. These loops are crucial for understanding the dynamic behaviour of complex systems and can be categorised into two main types: reinforcing (amplifying the effects within a loop) or balancing (stabilising the system by counteracting changes) [17]. With this, CLDs contribute to the understanding of how different elements in a system interrelate, often revealing unintended consequences and dynamic behaviour over time.

**Figure 1:**
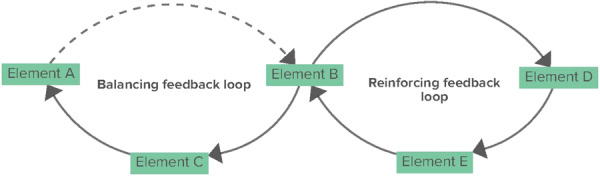
An illustration of a simple CLD and its output. Solid arrows indicate a positive relationship between elements, and dashed arrows indicate a negative relationship between elements. The CLD yielded two feedback loops, one balancing and one reinforcing feedback loop.

Within the CLD, elements are connected through arrows, including polarity. Solid arrows indicate a positive relationship between elements, meaning that elements move in the same direction (e.g., if the element increases, the connected element also increases). Dashed arrows indicate a negative relationship between elements, meaning that elements move in the opposite direction (e.g., if the element decreases, the connected element increases). Figure 1 gives an overview of a simple CLD, including elements connected through positive and negative relationships, resulting in two feedback loops.

The CLD generated in this study was developed using GMB, which is a participatory qualitative research method that systematically analyses complex problems through an iterative dialogue and collaborative modelling of system dynamics [15, 18]. To improve the transferability of the findings, the GMB sessions were guided by Scriptapedia, an online resource for conducting GMB practices [18].

The Centre for Clinical Expertise of the National Institute for Public Health and the Environment concluded that this study did not fall under the Dutch Medical Research Involving Human Subjects Act (WMO). Therefore, a formal ethical approval was deemed not to be necessary.

### 2.2 Participant Recruitment

This study was conducted in collaboration with the National Institute for Public Health and the Environment, the regional public health office of Groningen, the municipality Het Hogeland, and its corresponding local institution for social health services, Mensenwerk Hogeland. Het Hogeland is a rural municipality located in the Northern Netherlands with approximately 47,000 residents. Of these, 26% are adults above the age of 65 years, which is comparable to other rural municipalities in the Netherlands [19]. The municipality faces challenges typical of such ‘peripheral’ areas, including difficulties in providing adequate healthcare, social support and transportation. Therefore, the municipality is focusing on making the region more attractive and accessible to older adults, recognising the need for services and facilities that cater to an ageing population [20].

Participants were recruited through three community centres facilitated and coordinated by Mensenwerk Hogeland. These community centres are designed to foster social interaction and support residents, especially older adults living in the area. As they serve a broad and diverse population of older adults, these centres were considered feasible locations to recruit participants.

The initial contact with potential participants was laid out by the coordinator of the community centres, who had been briefed on the purpose of the study and inclusion criteria by the research team. A specific criterion was to include participants with a diversity in age, starting from 55 years old. The rationale for including adults from the age of 55 years was to capture diverse perspectives on vitality, recognising that transitions into later life may shape how individuals perceive their vitality [16]. Other criteria were that participants had to speak Dutch and were able to participate in all three GMB sessions. Participants who agreed to take part in the study received an information letter via email containing practical details. At the start of the first session, the study's purpose and details were reiterated, and participants were asked to provide written informed consent.

### 2.3 Procedure

**Figure 2:**
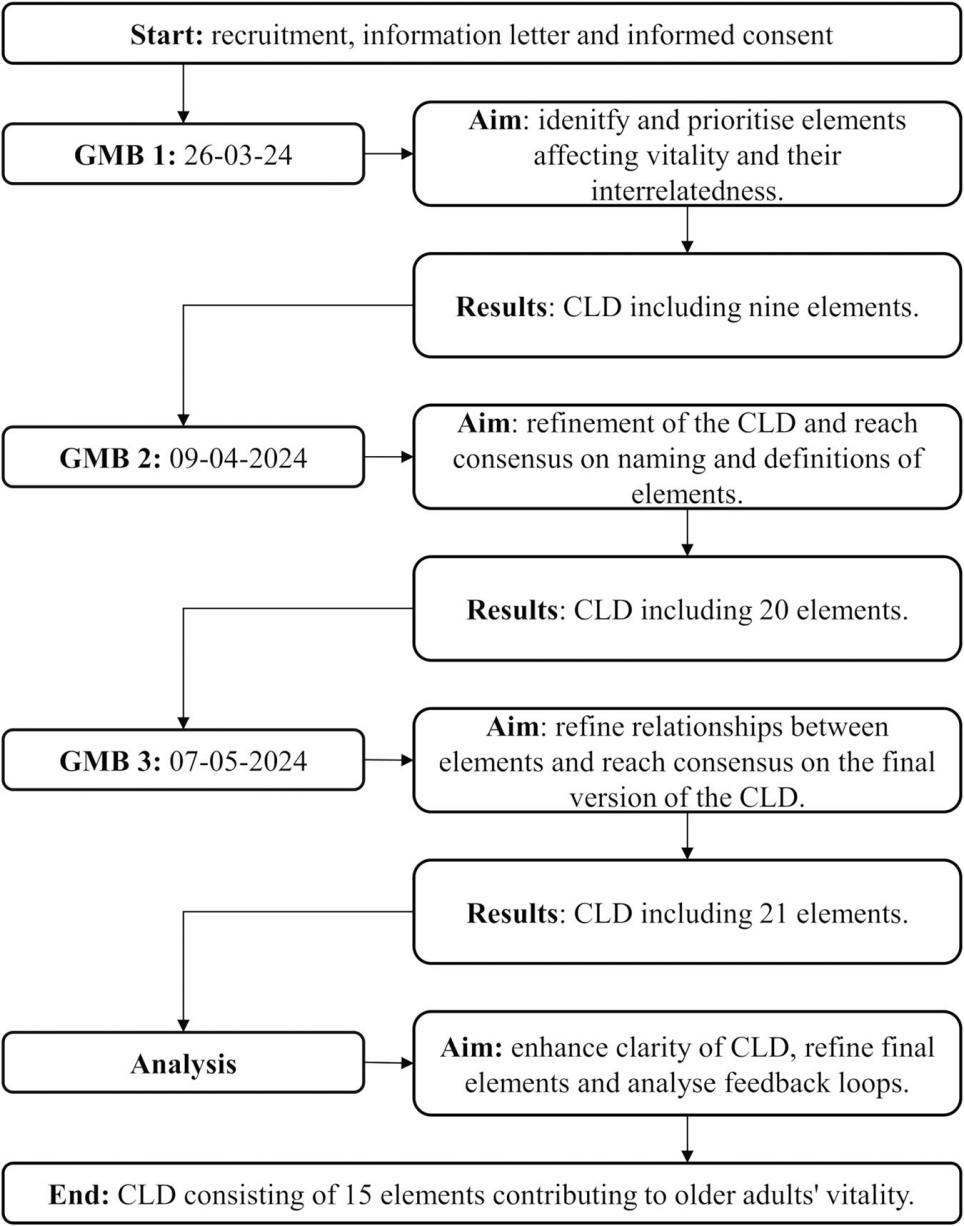
A schematic overview of GMB sessions, aims and results.

Figure 2 gives a schematic overview of the GMB sessions. To study the elements that influence older adults' vitality, we took a ‘blank slate’ approach. This means that the GMB sessions started open‐ended, and data on the elements that drive older adults' vitality was collected in an inductive manner.

All GMB sessions took place in local community centres. The researchers prepared each session with a detailed script on tasks, time management and the session's goals. During the sessions, notes were taken by a member of the research team. Besides, as a backup, sessions were recorded with an audio recorder. After each session, the research team debriefed, sharing initial impressions. A report was drafted to summarise the outcomes of each session. These reports were sent to participants before the following session. The following sections elaborate on the stepwise data collection through three GMB sessions.

### 2.4 GMB Session 1

The first GMB session, held in March 2024, aimed to identify and prioritise elements affecting vitality and their interrelatedness. In total, 12 participants joined the session. Because this was the first time participants and researchers met, the sessions started with a lunch to get acquainted. Getting acquainted was done to build rapport, contributing to a safe environment in which participants felt free to share their thoughts and opinions [21]. Then, participants were introduced to systems thinking and the purpose of the session. We introduced vitality as a state of ‘being able to do what you want to do’. This broad definition aligns with the idea that vitality enables people to live in alignment with their goals and personal desires while dealing with challenges when growing older [6]. We chose this broad definition to leave room for participants' interpretations of vitality.

After the general introduction, participants wrote down vitality‐related elements on sticky notes. These were consolidated by the researchers and discussed with the whole group to clarify the meaning of the elements and refine element names if needed. Then, participants prioritised elements through voting. The more votes an element got, the more relevant the element was considered.

During the last part of the session, participants were divided into two groups of six participants each. Facilitated by a member of the research team, the groups discussed the top nine elements. We decided on the top nine because the tenth element got substantially fewer votes than the first nine elements. The remaining elements were ‘parked’ to be discussed in the second GMB session. Participants mapped the top nine elements in a CLD to visualise how they were related to one another with arrows. Polarity was added to indicate the directions of relationships between elements. The session ended with a plenary discussion on the CLDs created by both groups.

After the session, the researchers conducted a debrief. Then, the two CLDs were combined into one CLD. This was done in the program Vensim PLE (version 10.1.0). The research team also revised the naming of some elements to align the exact formulation with the criteria of system dynamic maps. These elements should be neutral and quantifiable [17]. For instance, ‘Physical activity/sports’ was renamed to ‘Daily physical activity’. Besides, overlapping elements were grouped to avoid redundancy. If elements needed further refinement, they were written down to discuss in the next session with participants. Finally, a report summarising the outcomes of the first session was drafted and shared with the participants before the next session.

### 2.5 GMB Session 2

The second session took place 2 weeks after the first session, in April 2024, and was attended by 11 out of 12 participants who attended the first GMB session. This session aimed to refine the combined CLD and reach a consensus on the naming and definitions of elements.

The session started with a recap of the last session and a presentation of the combined CLD. Reflecting on the results from the first session, participants recognised that the naming of some elements included in the CLD was unclear. These elements were discussed and renamed. For instance, one element in the CLD was initially called ‘mental health’. Participants suggested renaming it to ‘mental well‐being’ to better reflect a broader range of factors contributing to a positive sense of self and feeling well. The researchers added adjustments in the naming of elements directly to the CLD.

In the second part of the session, the focus was on refining the CLD, reviewing the ‘parked’ elements and reaching a consensus on the elements included in the CLD. Participants were divided into two separate groups, one of six participants and one of five participants. Group discussions were facilitated by a researcher, who emphasised that the goal was to map the most relevant elements contributing to vitality rather than drawing out the whole system. The group made adjustments by adding or removing elements and refining their associated relationships as necessary.

Lastly, adjustments and additions made to the CLDs were discussed in the whole group. Any differences that arose were discussed and adjusted to achieve consensus. In summary, a total of 11 elements were added to the CLD. The research team made these additions on the spot, displayed on a large screen.

Once again, the researchers debriefed immediately after the session to discuss the additions made to the CLD. Leading up to the third session, researchers optimised the CLD in Vensim PLE (version 10.1.0). Researchers observed some ambiguities in the naming of elements and documented these for discussion with participants during the third session. A report on the outcomes of the second session was drafted and shared with the participants before the start of the third session.

### 2.6 GMB Session 3

The third and last session took place 3 weeks after the second session, at the beginning of May 2024. The session was attended by 10 out of 12 participants who joined the first GMB session. The goal of the session was to refine relationships between elements and reach a consensus on the final version of the CLD.

Facilitated by a member of the research team, participants explored the relationships between elements and different loops in the CLD. To enhance discussion, researchers grouped related elements into three clusters. The cluster ‘Psychological and emotional elements’ categorises elements related to emotional and psychological aspects of vitality. The cluster ‘Social connections and interpersonal support’ categorises elements related to social relationships and interactions. The cluster ‘Lifestyle and daily habits’ categorises elements related to nutrition and physical activity. In groups of 3–4 people, participants discussed the elements and relationships of each cluster. If adjustments were made, they were added directly to the CLD by the facilitator.

Next, adjustments were discussed and checked by all participants. This established the foundation for the final CLD. The session was concluded by thanking the participants for their time and effort and handing them a €50 gift voucher. After the third GMB session, a report including the insights from the last sessions was sent to all participants.

### 2.7 Analysis

The creation of the CLD was an iterative process. While participants built the CLD during the GMB sessions, researchers refined the CLD based on their expert knowledge in between sessions. The adjustments were made by the researchers and focused on re‐naming the elements, eliminating illogical connections and incorporating missing links. In the consecutive session, the adjustments made by the research team were discussed with the participants to facilitate member checking. This way, the CLD was built through co‐creation and yielded an overview of the elements of older adults' vitality and how they interrelate with each other.

In the final phase of the analysis, the CLD was transferred from Vensim to the online modelling tool ‘Kumu’. This was done to enhance the visual quality of the CLD and to analyse feedback loops. In this process, the definitions and naming of elements were translated into English, and the CLD was visually rearranged to enhance clarity. For example, elements that belonged to the same cluster were assigned the same colour. Furthermore, feedback loops detected by Kumu were compared with the discussion on loops by participants. In the results, we describe these feedback loops, emphasising system dynamics, relationships between elements, and loops that connect multiple clusters.

## 3 Results

In total, 12 older adults participated in our study. The group of participants consisted of five men and seven women. The age of participants ranged between 57 and 89 years, with a mean age of 73 years. Four participants were aged between 57 and 70 years, five participants were between 70 and 80 years, and three were above 80 years.

**Figure 3:**
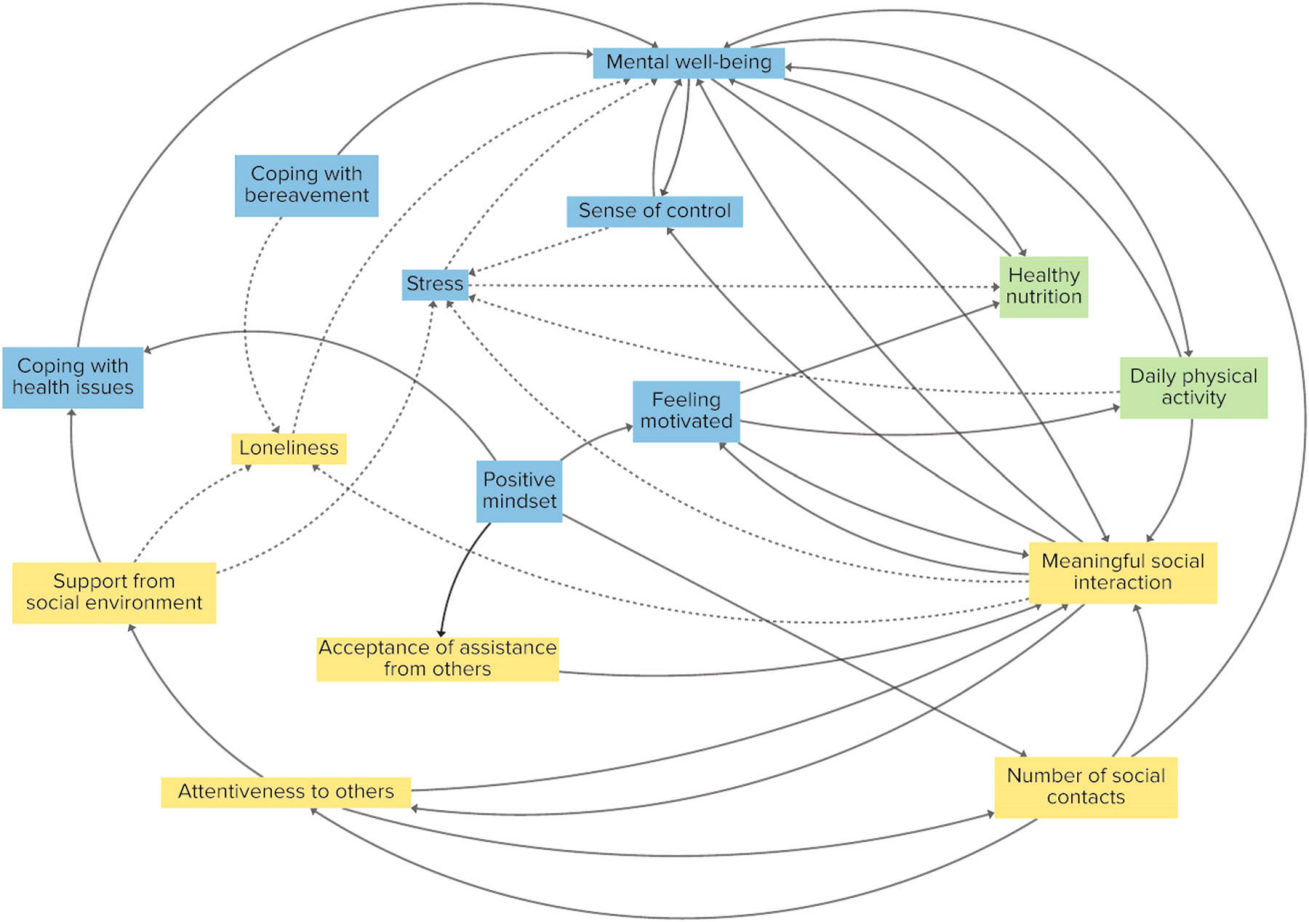
The final CLD, consisting of 15 elements that contribute to older adults' vitality. The blue elements belong to the cluster ‘Psychological and emotional elements’. The yellow elements belong to the cluster ‘Social connections and interpersonal support’. The green elements belong to the cluster ‘Lifestyle and daily habits’. Solid arrows indicate a positive relationship between elements, and dashed arrows indicate a negative relationship between elements.

Figure 3 displays the final CLD, consisting of 15 elements contributing to older adults' vitality. The blue elements belong to the cluster ‘Psychological and emotional elements’, consisting of seven elements. The yellow elements belong to the cluster ‘Social connections and interpersonal support’, consisting of six elements. The purple elements belong to the cluster ‘Lifestyle and daily habits’, consisting of two elements. Table 1 reports the elements and their definitions.

**Table 1:** Overview of elements and their definitions in the final CLD.

Element	Cluster	Definition
Support from the social environment	Social connection and interpersonal support	Emotional and practical assistance, provided by individuals who are part of someone's social circle, such as family members, close friends and neighbours.
Number of social contacts	Social connection and interpersonal support	Any social connection or encounter with others, regardless of depth or quality.
Meaningful social interaction	Social connection and interpersonal support	Refers to deeper, more emotionally significant engagements with others, often involving a sense of connection, understanding, empathy and mutual respect.
Attentiveness to others	Social connection and interpersonal support	The quality of being mindful, observant of, and considerate towards the needs, feelings and perspectives of other people.
Loneliness	Social connection and interpersonal support	A distressing emotional response to perceived isolation or lack of companionship, characterised by a discrepancy between the desired and actual social interactions.
Acceptance of assistance from others	Social connection and interpersonal support	Being open to receiving help, support or advice from other people, such as family members or (health)professionals.
Coping with bereavement	Psychological and emotional elements	Navigating the period of grief and mourning following the death of a loved one constructively and adaptively to gradually find a new normal.
Mental well‐being	Psychological and emotional elements	A holistic sense that refers to a state of psychological and emotional health characterised by a positive sense of self, life satisfaction, emotional resilience and the ability to cope with challenges.
Sense of control	Psychological and emotional elements	The perception or belief that one has the power to influence or direct events, outcomes and actions within their environment.
Stress	Psychological and emotional elements	The psychological and physiological response to demands or pressure that are perceived as exceeding an individual's ability to cope.
Coping with health issues	Psychological and emotional elements	The various strategies and actions taken to cope with health issues related to growing older.
Feeling motivated	Psychological and emotional elements	An internal drive that initiates, directs and sustains goal‐oriented behaviours.
Positive mindset	Psychological and emotional elements	A mental attitude that focuses on looking for the good in situations, expecting favourable outcomes and approaching challenges with optimism.
Healthy nutrition	Lifestyle and daily habits	Foods that fuel the body in a healthy, nutritious way to maintain energy levels and manage or prevent health issues through a balanced and nutrient‐rich diet.
Daily physical activity	Lifestyle and daily habits	Movement that helps maintain or improve physical health and functional abilities, including a wide range of activities such as structured exercise to more routine movements like gardening or household chores.

### 3.1 Overall System Dynamics

The CLD displays the diversity of elements that contribute to older adults' vitality and their interrelatedness. With this, participants express that vitality is a composition of physical, social and psychological dimensions. In total, 41 feedback loops were detected. Of these, 21 loops include elements from all three clusters, 15 loops include elements from two clusters, and 5 loops are within a cluster. All loops are reinforcing feedback loops.

### 3.2 Psychological and Emotional Elements

This cluster encompasses psychological and emotional elements related to older adults' vitality. The seven elements that belong to this cluster are mental well‐being, stress, feeling motivated, a positive mindset, a sense of control, coping with bereavement and coping with health issues.

**Figure 4:**
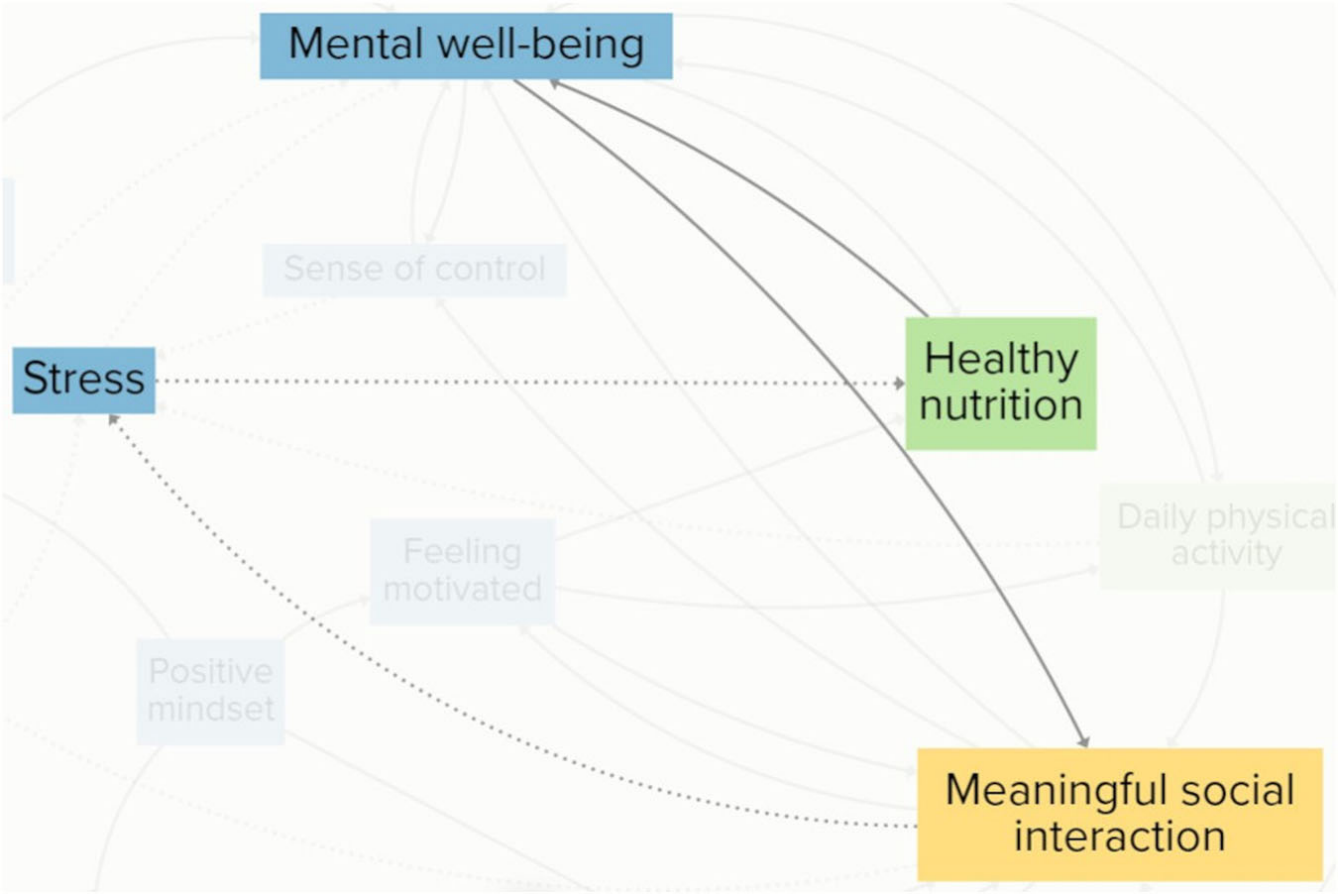
An extract from the CLD displaying a reinforcing feedback loop between stress, healthy nutrition, mental well‐being and meaningful social interaction. Solid arrows indicate a positive relationship between elements, and dashed arrows indicate a negative relationship between elements.

For participants, psychological and emotional elements were intrinsic drivers of vitality, highly linked to elements grouped in the other clusters. They emphasised that often psychological and emotional elements were influenced by circumstances beyond their control. Some of these were inherent to the process of ageing. These were included as elements in the CLD, such as coping with bereavement and coping with health issues. Figure 4 illustrates a reinforcing feedback loop, showing how rising stress can trigger a cascade of interconnected effects that ultimately diminish older adults' vitality. Participants explained that when their stress levels rose, they sought solace in eating unhealthy food, decreasing their healthy nutrition intake. A decrease in healthy nutrition negatively impacted their mental well‐being, because they felt worse about themselves. They explained that a decline in mental well‐being could lead to social withdrawal, reducing meaningful social interactions. All together, the loop demonstrates how escalating stress can set off a self‐reinforcing cycle, affecting multiple dimensions of vitality.

### 3.3 Social Connections and Interpersonal Support

This cluster centres on social relationships and the quality of interactions with others. The six elements that belong to this cluster are: attentiveness to others, acceptance of assistance from others, loneliness, number of social contacts, meaningful social interactions and support from the social environment.

**Figure 5:**
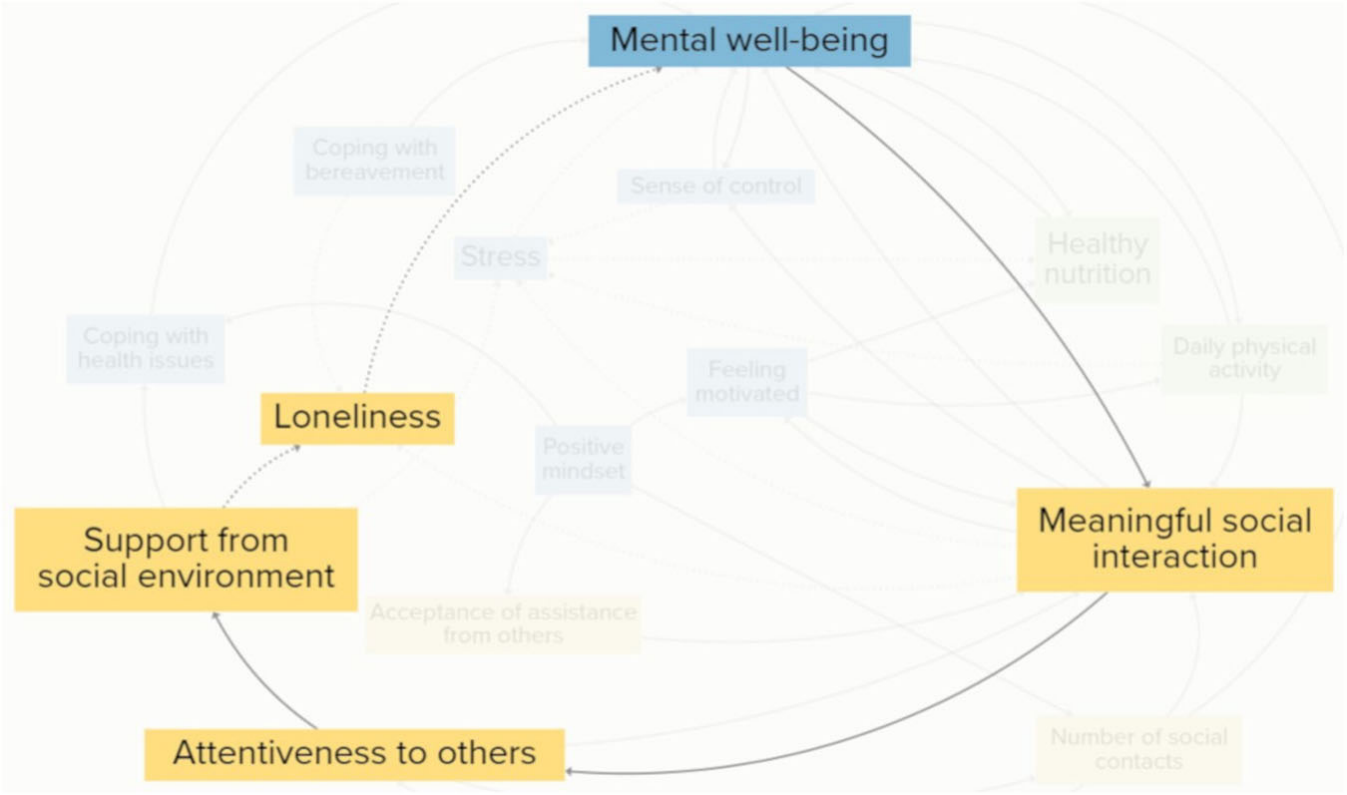
An extract from CLD displaying a reinforcing feedback loop between meaningful social interaction, attentiveness to others, support from social environment, loneliness and mental well‐being. Solid arrows indicate a positive relationship between elements, and dashed arrows indicate a negative relationship between elements.

Participants emphasised the importance of social relationships for their vitality. They explained that when they engaged in social relationships, other elements related to their vitality flourished. Figure 5 illustrates a reinforcing feedback loop on the mitigating effects of social relationships. The feedback loop demonstrates the positive effect of meaningful social interactions, attentiveness to others and support from the social environment on loneliness, leading to an increase in mental well‐being. According to participants, an increase in meaningful social interactions naturally led to greater attentiveness to others. This was because meaningful social interactions fostered a deeper sense of connection, which encouraged them to be more curious and sensitive to the needs of others. They perceived the heightened attentiveness as a catalyst for positive social exchanges, resulting in an increase in support from the social environment. This was linked to a decrease in loneliness because social support had buffering effects on life challenges, causing loneliness, such as losing a loved one or having to move houses due to age. Participants related a decrease in loneliness to an increase in mental well‐being, which in itself was related to an increase in meaningful social interaction. Together, this reinforcing feedback loop visualises the buffer of social connections and interpersonal support against the adverse effects of loneliness.

### 3.4 Lifestyle and Daily Habits

This cluster groups elements related to lifestyle and daily habits, including healthy nutrition and daily physical activity. Healthy nutrition is linked to the cluster of psychological and emotional elements. Daily physical activity is linked to the cluster of psychological and emotional elements and elements that belong to the cluster of social connections and interpersonal support.

**Figure 6:**
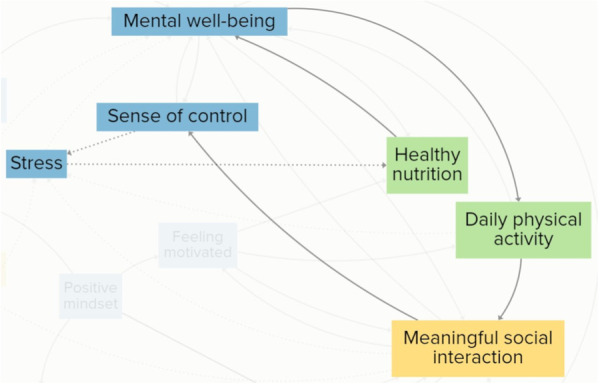
An extract from the CLD displaying a reinforcing feedback loop between daily physical activity, meaningful social interaction, sense of control, stress, healthy nutrition and mental well‐being. Solid arrows indicate a positive relationship between elements, and dashed arrows indicate a negative relationship between elements.

Participants perceived the elements that belong to lifestyle and daily habits as a motivator of various mechanisms that contributed to their vitality. They argued that the positive effects of daily physical activity and healthy nutrition extended far beyond physical health improvements. A reinforcing feedback loop that encompasses the positive effect of daily physical activity and healthy nutrition on vitality is illustrated in Figure 6. This loop displays the impact of daily physical activity on meaningful social interaction, a sense of control, stress, healthy nutrition and mental well‐being. According to the participants, daily physical activity created various opportunities for meaningful social interaction. One example was that physical activities were often performed in a group setting, providing a natural environment to meet (new) people. Another example was the social benefits of a stroll through the neighbourhood, creating opportunities to connect with neighbours and creating a sense of community. This way, participants felt that daily physical activity enriched their social lives, causing an increase in meaningful social interaction. Participants related the increase in meaningful social interaction to an increase in a sense of control because meaningful social interactions provided a setting in which they could make their own decisions. For example, choosing what kind of social activities they want to engage in. Participants expressed that an increase in a sense of control decreased their stress levels. Such a decrease in stress was related to positive effects on the consumption of healthy nutrition, as participants explained that they were less inclined to consume comfort food. Such improvements in their diet made them feel better about themselves, increasing their mental well‐being. Closing the loop, participants related an increase in mental well‐being to increased energy, finding the willpower and motivation to engage in daily physical activity. Participants emphasised that daily physical activity and healthy nutrition could function as leverage points to enhance their vitality.

## 4 Discussion

To the best of our knowledge, this is the first participatory study to map elements that contribute to older adults' vitality from a system's perspective. Our study yielded 15 elements that contribute to older adults' vitality, according to the older adults themselves. These were categorised into three clusters: psychological and emotional elements, elements related to social connections and interpersonal support and elements related to lifestyle and daily habits. The CLD displays the diversity of elements contributing to older adults' vitality and their dynamic interplay, revealing the interdisciplinary composition of physical, social and psychological dimensions that shape older adults' vitality.

Our findings underscore that older adults' vitality encompasses more than being physically and mentally fit; it is shaped by the interaction of multiple domains that collectively contribute to an individual's experience of vitality. This integrated view of vitality aligns closely with the concept of Positive Health, which offers a broader view on health and recognises that health involves resilience, the ability to cope with stress, and the capacity to maintain a meaningful and active life [22]. In line with Positive Health, the CLD displays that vitality occurs through an interplay of various elements. An example is the buffering effect of healthy nutrition and daily physical activity. This is not surprising, as a healthy lifestyle is a well‐known protective element of vitality [23]. Our CLD adds to this understanding by visualising how daily physical activity and healthy nutrition can accelerate vitality through their interrelatedness with mental well‐being and meaningful social interactions.

Participants emphasised the pivotal role of social connections and interpersonal support in sustaining their vitality. This finding aligns with a well‐established body of research linking supportive social environments to improved health outcomes and enhanced well‐being in older populations [7, 21]. Our study highlights an important dynamic of give‐and‐take in social relationships, which can be understood as reciprocal exchanges of care, emotional support and practical assistance, reinforcing social networks over time [24]. As interest in interventions promoting social connectedness among older adults grows, understanding the reinforcing nature of these relationships is essential for fostering long‐term vitality. This perspective shifts the focus from individual interactions to broader systemic processes that cultivate resilient, supportive communities for older adults.

The exclusive presence of reinforcing feedback loops in our CLD indicates that participants predominantly conceptualised vitality as a product of cumulative, self‐reinforcing processes. One likely explanation for this outcome is that all participants identified as vital, meaning that barriers to vitality, for example, depressive symptoms or functional declines [6, 7], were not central to their daily experiences. Consequently, discussions primarily focused on the preconditions and enhancing factors of vitality, rather than on challenges or hindrances. This focus may reflect an optimism bias, potentially overlooking balancing loops that could counteract or constrain vitality [23]. To develop a more nuanced understanding, we recommend that future research engage with individuals who hold a broader range of experiences, including those who may not view themselves as vital. This approach would help identify potential barriers and balance feedback mechanisms that contribute to a fuller picture of vitality dynamics.

This study advances the understanding of how multiple, interconnected elements shape older adults' vitality. It shows that older adults' vitality emerges from a dynamic interplay between individual behaviours and capacities and broader systemic conditions. While self‐directed actions, such as engaging in physical activity, eating healthy and initiating social contact, are part of older adults' vitality, the impact of these actions is shaped by factors beyond individual control. This interaction between individual and systemic elements challenges reductionist approaches that focus on isolated determinants of vitality. Instead, our findings point to the need for comprehensive, multidisciplinary strategies that integrate behavioural interventions with structural and community‐level initiatives. For example, drawing on the CLD, we suggest that environments that foster social connections are essential for supporting older adults' vitality. However, creating such environments requires a multidimensional approach beyond merely providing social opportunities. It must also stimulate an individual's capacity and willingness to engage in meaningful relationships.

One of the key strengths of this study was the use of participatory‐led modelling. By actively engaging older adults in the research process, the CLD authentically reflects their lived experiences, priorities and contextual nuances, rather than being shaped solely by external or academic perspectives. Such meaningful involvement leads to richer, more applicable insights and fosters a sense of ownership and empowerment among participants [25]. Furthermore, the use of continuous member checking throughout the research process, which is a recognised strategy to enhance the credibility and validity of qualitative research, allowed participants to confirm or clarify the interpretations of the researchers, thus reducing potential biases [26]. Lastly, the research team consisted of researchers with various backgrounds, bringing diverse viewpoints and ensuring that interpretations were not dominated by one perspective. This type of triangulation enhanced the richness of the data collected and ensured that the participant‐led modelling process was carried out with sensitivity and rigour. Altogether, the study yielded information that informs future research and broadens interdisciplinary understanding of vitality. Through its visualisation of the dynamic interaction between diverse elements, the CLD is a valuable tool that facilitates the development of integrated policies and programmes to promote vitality among older adults.

While using GMB to create a CLD on how older adults perceive vitality offers valuable insights, several limitations should be acknowledged to better understand the scope and implications of our findings. First, participants considered themselves vital, potentially excluding challenges faced by less vital people. Furthermore, due to the sample's relative homogeneity, our study might not capture differences in perceptions on vitality related to living environments (e.g., rural vs. urban settings), as well as diverse socio‐economic and cultural backgrounds. Moreover, the majority of our participants were aged 70 and above. With this, the results of our study might not fully capture the perspectives of younger older adults who are navigating different transitions and priorities in life [27]. This selectivity limits the external generalisability of our findings. Another limitation is that the CLD represents a conceptual simplification and, by necessity, omits certain variables or interactions pertinent to older adults' vitality in various contexts. Additionally, the study focusing on individual perceptions may underemphasise macro‐level influences. This could explain the strong representation of psychological and emotional elements and elements related to social connections and interpersonal support, while structural factors, such as healthcare access, are scarcely noted by participants. Finally, the CLD offers a static view and does not capture potential changes in the system, limiting its real‐world applicability [28]. Thus, the CLD should be used primarily as a discussion tool rather than a definitive model. To make these findings actionable, we recommend using design thinking. Design thinking is a systems approach used to understand end users' needs through iteratively testing solutions [29]. These methods are highly effective in developing innovative solutions in complex and dynamic environments [30].

## 5 Conclusions

In conclusion, this study provides valuable insights into the complex interplay of elements influencing older adults' vitality, highlighting the critical roles of psychological, social and lifestyle‐related elements. By visualising these elements through a CLD, we have demonstrated how vitality is sustained by reinforcing feedback loops, emphasising its dynamic nature. The participatory‐led approach yielded co‐produced insights that inform public health strategies, underscoring the need for comprehensive, multidisciplinary approaches to promote vitality in later life. Such approaches must offer social opportunities and foster individuals' capacity and motivation to engage in meaningful social relationships.

## Author Contributions

**Djoeke Besselink:** conceptualisation (equal), methodology (equal), software (equal), data curation (equal), investigation (equal), validation (equal), formal analysis (lead), resources (equal), visualisation (lead), writing – original draft (lead), writing – review and editing (equal). **Fons van der Lucht:** conceptualisation (equal), methodology (equal), software (equal), data curation (equal), investigation (equal), validation (equal), formal analysis (supporting), supervision (equal), funding acquisition (equal), resources (equal), writing – review and editing (equal). **Lisa Barsties:** conceptualisation (equal), methodology (equal), software (equal), data curation (equal), investigation (equal), validation (equal), resources (equal), writing – review and editing (equal). **Martine Jansen‐van der Vliet:** conceptualisation (equal), methodology (equal), software (equal), data curation (equal), investigation (equal), validation (equal), resources (equal), writing – review and editing (equal). **Thomas G. Kuijpers:** conceptualisation (equal), methodology (equal), software (equal), data curation (equal), investigation (equal), validation (equal), resources (equal), writing – review and editing (equal). **Lidwien Lemmens:** conceptualisation (equal), methodology (equal), software (equal), data curation (equal), investigation (equal), validation (equal), resources (equal), writing – review and editing (equal). **Evelyn J. Finnema:** validation (equal), supervision (equal), writing – review and editing (equal). **Saskia W. van den Berg:** conceptualisation (equal), methodology (equal), software (equal), data curation (equal), investigation (equal), validation (equal), supervision (equal), funding acquisition (equal), project administration (lead), resources (equal), writing – review and editing (equal).

## Conflicts of Interest

The authors declare no conflicts of interest.

## Data Availability

The data that support the findings of this study are available upon request from the corresponding author. The data are not publicly available due to privacy or ethical restrictions.
